# Fermented Wild Ginseng by *Rhizopus oligosporus* Improved l-Carnitine and Ginsenoside Contents

**DOI:** 10.3390/molecules25092111

**Published:** 2020-04-30

**Authors:** Ganghee Lee, Thi Thanh Hanh Nguyen, Tae Yun Lim, Juho Lim, Byeongsu Park, Seonmin Lee, Il-Kyoon Mok, Kunal Pal, Sangyong Lim, Doman Kim

**Affiliations:** 1 Graduate School of International Agricultural Technology, Seoul National University, Pyeongchang-gun, Gangwon-do 25354, Korea; darklue@naver.com (G.L.); juholim@snu.ac.kr (J.L.); mpbs91@snu.ac.kr (B.P.); luck1035@snu.ac.kr (S.L.); 2 Institute of Food Industrialization, Institutes of Green Bio Science & Technology, and Center for Food and Bioconvergence, Seoul National University, Pyeongchang-gun, Gangwon-do 25354, Korea; hara2910@snu.ac.kr (T.T.H.N.); mokpodong@snu.ac.kr (I.-K.M.); 3 Department of English, Hongik University, Seoul 04066, Korea; tylim27@hongik.ac.krU; 4 Department of Biotechnology and Medical Engineering, National Institute of Technology, Rourkela 769008, India; kpal.nitrkl@gmail.com; 5 Radiation Research Division, Korea Atomic Energy Research Institute, Jeongeup 56212, Korea; saylim@kaeri.re.kr

**Keywords:** fermentation, ginsenoside, l-carnitine, *Panax ginseng*, *Rhizopus oligosporus*

## Abstract

We conducted this study to investigate the beneficial effects of *Rhizopus oligosporus* fermentation of wild ginseng on ginsenosides, l-carnitine contents and its biological activity. The *Rhizopus oligosporus* fermentation of wild ginseng was carried out at 30 °C for between 1 and 14 days. Fourteen ginsenosides and l-carnitine were analyzed in the fermented wild ginseng by the ultra high pressure liquid chromatography–mass spectrometry (UPLC–MS) system. Our results showed that the total amount of ginsenosides in ginseng increased from 3274 to 5573 mg/kg after 14 days of fermentation. Among the 14 ginsenosides tested, the amounts of 13 ginsenosides (Rg1, Rb2, Rb3, Rc, Rd, Re, Rf, Rg2, Rg3, Rh1, compound K, F1 and F2) increased, whereas ginsenoside Rb1 decreased, during the fermentation. Furthermore, l-carnitine (630 mg/kg) was newly synthesized in fermented ginseng extract after 14 days. In addition, both total phenol contents and DPPH radical scavenging activities showed an increase in the fermented ginseng with respect to non-fermented ginseng. These results show that the fermentation process reduced the cytotoxicity of wild ginseng against RAW264.7 cells. Both wild and fermented wild ginseng showed anti-inflammatory activity via inhibition of nitric oxide synthesis in RAW264.7 murine macrophage cells.

## 1. Introduction

Ginseng (*Panax ginseng* Meyer) has been considered a popular medicinal herb in Asian countries for over 2000 years and is used as a traditional medicine and health-enhancing supplement similar to a tonic and prophylactic [1]. *P. ginseng* is classified into wild or cultivated types according to the different seeding methods [2,3,4]. Ginseng contains ginsenosides and exhibits multiple pharmacological activities such as anti-inflammatory, antihypertensive, antidiabetes, antistress, anticancer, antioxidant and neuroprotection effects [5,6,7,8,9]. Based on their aglycone structure, ginsenosides are categorized into three groups: oleananes, protopanaxadiols (PPD) and protopanaxatriols (PPT) [4,10,11]. The PPD- and PPT-type ginsenosides carry different sugars linked to C-3 and C-20 in the aglycone PPD and to C-6 and C-20 in the aglycone PPT [10]. The biological activity of ginseng is affected by deglycosylation of the main ginsenosides to form minor ginsenosides, which have smaller size, higher bioavailability and higher permeability [12,13]. For example, the anti-inflammatory and antitumor effects of compound K transformed from ginsenosides (Rb1, Rb2 and Rc) were improved [14,15]. Therefore, numerous studies have focused on the synthesis of minor ginsenosides by hydrolyzing the sugar residues linked to the C-3, C-6 and C-20 positions of the major ginsenosides [16,17] through various procedures such as heating and acid treatment [18,19], an enzymatic method (β-glucosidase) [20,21] and microbial fermentation [16,17,22,23,24]. In microbial fermentation, most of the microbial strains such as *Lactobacillus plantarum*, *Lactobacillus delbrueckii*, *Bifidobacterium longum* and *Rhizopus stolonifera* were used to produce minor ginsenosides from the main ginsenosides by deglycosylation using β-glucosidase [16,23], and to improve phenolic contents and antioxidant activities [22].

*Rhizopus oligosporus* (*R. oligosporus*), a fungus found predominantly in fermented soybean products such as Indonesian tempeh, hydrolyzes protein into amino acids and small peptides by various enzymes such as lipase, amylase, protease and glucoamylase, and synthesizes l-carnitine and γ-aminobutyric acid (GABA) in soybean, buckwheat and quinoa [25,26,27]. l-carnitine plays an important role in the transportation of fatty acids into the mitochondrial compartment for β-oxidation and subsequent energy production and antioxidant activity to prevent peroxidative damage [28,29]. It also produces carbohydrate-cleaving enzymes such as β-glucosidase, β-glucuronidase and xylanase to create polyphenols from carbohydrates-conjugated phenolic compounds during fermentation [30,31]. *R. oligosporus* had been used to increase phenolic contents and antioxidant activities of soybean, buckwheat and quinoa through fermentation [27,32]. However, increased l-carnitine contents in fermented ginseng associated with biochemical characterization have not been reported. In the present study, we selected *R. oligosporus* for fermentation of wild ginseng and determined the amount of l-carnitine as well as ginsenoside contents in the fermented wild ginseng. Furthermore, we investigated the total phenol contents as well as the effects of combination among phenolic compounds, ginsenosides and l-carnitine on antioxidant and anti-inflammatory activity via inhibition of nitric oxide synthesis in the RAW264.7 murine macrophage cells of fermented wild ginseng.

## 2. Results and Discussion

### 2.1. Changes in Ginsenoside Composition of Fermented Wild Ginseng

In this study, we evaluated the extraction yields in eight cases of wild ginseng, autoclaved wild ginseng and fermented wild ginseng after 1-, 3-, 5-, 7-, 10- and 14-day fermentation, as shown in Table 1. The extraction yields increased with increasing fermentation time from 21.2% (1-day fermentation) to 25.4% (14-day fermentation).

The ginsenoside composition of fermented ginseng after 0–14 days of fermentation is shown in Table 2. After autoclaving the ginseng extracts for sterilization and gelatinization, the amount of 10 ginsenosides (Rg2, Rg3, Rb1, Rb2, Rb3, Rc, Rh1, CK, F1 and F2) increased significantly while that of 4 ginsenosides (Rg1, Rd, Re and Rf) did not show significant change (*p* < 0.05). The increase of ginsenosides possibly resulted from liberation and breakdown of the cell matrix during thermal processing (autoclaving). These results were similar to those observed in a study by Hwang et al. (2014) [33].

**Figure 1:**
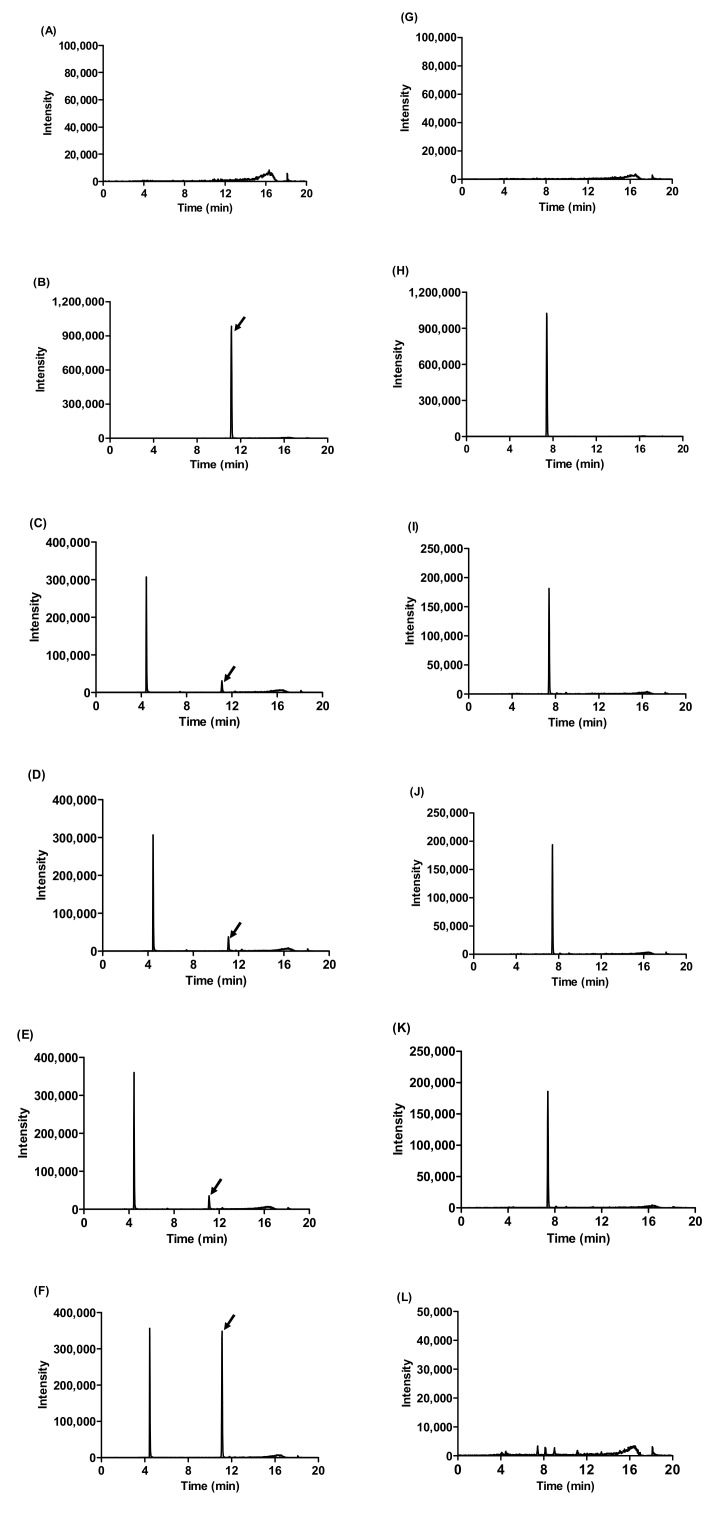
Chromatograms of ginsenoside Rd and Rb1 derived from the fermented wild ginseng extract using LC/MS. Detection of Rd ginsenoside: (**A**) blank (SIR: 969.7 *m*/*z*), (**B**) ginsenoside Rd standard (SIR: 969.7 *m*/*z*), (**C**) the wild ginseng extract (SIR: 969.7 *m*/*z*), (**D**) the autoclaved wild ginseng extract (SIR: 969.7 *m*/*z*), (**E**) 1 day (SIR: 969.7 *m*/*z*) and (**F**) 14-day (SIR: 969.7 *m*/*z*) of fermented wild ginseng extract. Detection of ginsenoside Rb1: (**G**) blank (SIR: 1132.0 *m*/*z*), (**H**) ginsenoside Rb1 standard (SIR: 1132.0 *m*/*z*), (**I**) the wild ginseng extract (SIR: 1132.0 *m*/*z*), (**J**) the autoclaved wild ginseng extract (SIR: 1132.0 *m*/*z*), (**K**) 1-day (SIR: 1132.0 *m*/*z*) and (**L**) 14-day (SIR: 1132.0 *m*/*z*) of fermented the wild ginseng extract.

After 14 days of fermentation, the amount of 13 ginsenosides (Rg1, Rg2, Rg3, Rb2, Rb3, Rc, Rd, Re, Rf, Rh1, F1, F2 and CK) increased (Table 2). Among them, the amount of ginsenoside Rd, initially 1280 ± 60 mg/kg in the wild ginseng and 2020 ± 370 mg/kg in the autoclaved ginseng, increased to 11,300 ± 840 mg/kg in the 14-day fermented wild ginseng, while Rb1, initially 4030 ± 540 mg/kg in wild ginseng and 7890 ± 1240 mg/kg in autoclaved ginseng, decreased to 400 ± 40 mg/kg in the 14-day fermented wild ginseng (Table 2 and Figure 1). Rb1 carries an additional sugar residue at the C-20 position with respect to Rd. Therefore, it is possible that the major ginsenoside Rd is converted mainly from Rb1. Noh et al. (2009) reported that β-glycosidase from *Sulfolobus solfataricus* can hydrolyzed from Rb1 to Rd [34]. In addition, phenolic, flavonoids and ginsenoside compounds occur as soluble conjugates and insoluble forms, covalently bound to sugar moieties or cell-wall structural components [35,36]. Moreover, *R. oligosporus* is known to produce β-glucosidase, β-glucuronidase and xylanase through the degradation of the cell-wall matrix [31]. Through the hydrolysis of glycosidic bonds by these enzymes, the ginsenoside compounds are probably released from bound ginsenosides, and aglycone forms during fermentation [31]. Recently, many studies have demonstrated that minor ginsenosides (Rd, Rg3 and compound K) possess even more remarkable pharmacological activity than major ginsenosides do. These minor ginsenosides are known to be produced through hydrolysis of sugar residues of major ginsenosides [12,14,15]. These results show that the concentrations of minor ginsenosides (Rg3, Rd and compound K) in wild ginseng extracts were increased by *R. oligosporus* fermentation.

### 2.2. Amount of l-Carnitine in Fermented Wild Ginseng

**Figure 2:**
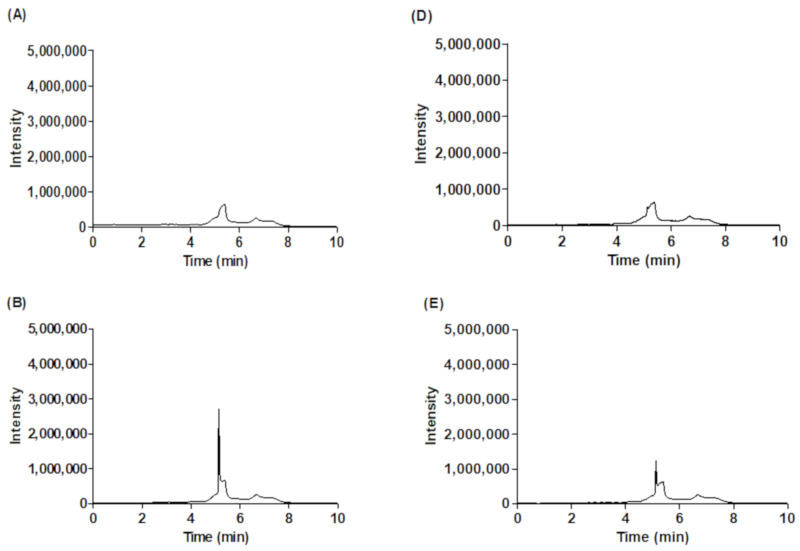

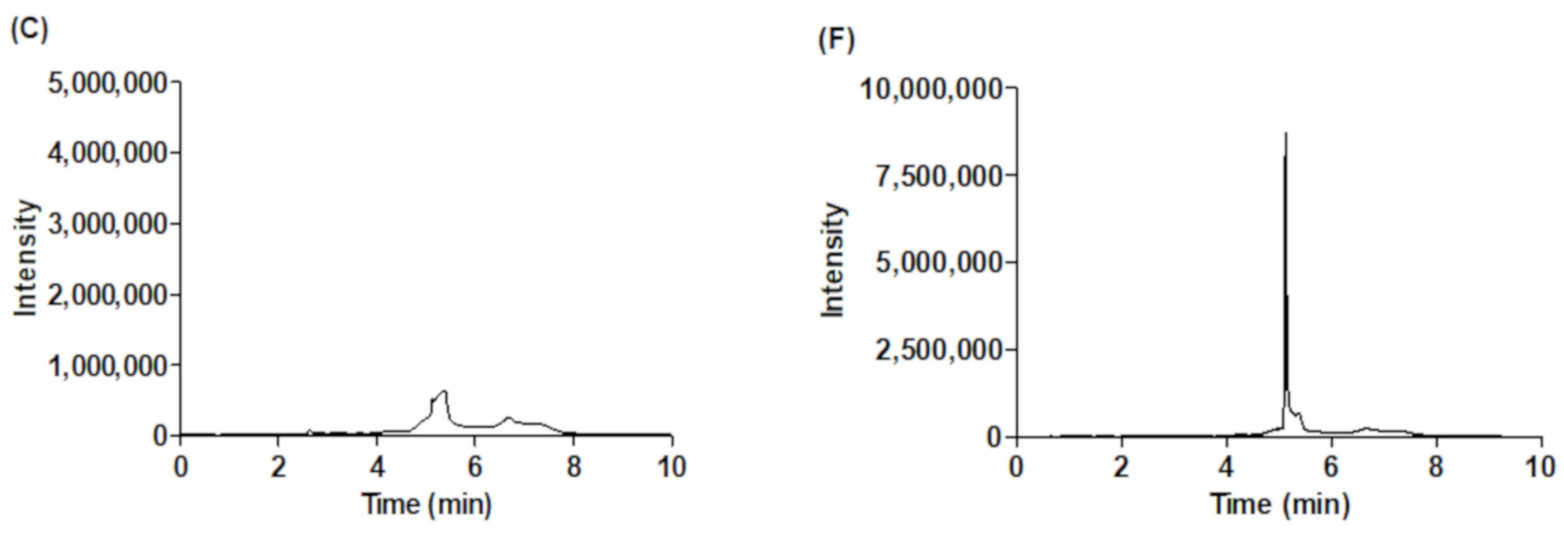
Chromatograms of l-carnitine derived from the fermented wild ginseng extract by LC/MS. (**A**) Blank (SIR: 162 *m*/*z*), (**B**) l-carnitine standard (SIR: 162 *m*/*z*), (**C**) the wild ginseng extract (SIR: 162 *m*/*z*), (**D**) the autoclaved wild ginseng (SIR: 162 *m*/*z*), (**E**) 1-day (SIR: 162 *m*/*z*) and (**F**) 14-day (SIR: 162 *m*/*z*) of fermented wild ginseng extract.

As shown in Figure 2, the peak of standard l-carnitine was eluted at 5.04 min in 1-day fermented ginseng and at 5.05 min in 14-day fermented wild ginseng. l-carnitine was not detected in the wild and autoclaved wild ginseng extracts but it was detected after 1-day fermentation (60 mg/kg; Table 3). The amount of l-carnitine in fermented wild ginseng increased from 60 mg/kg in 1-day fermentation to 630 ± 10 mg/kg in 14-day fermentation (Table 3). These results show that the l-carnitine contents increased in fermented wild ginseng as *R. oligosporus* hydrolyzed protein into amino acids and small peptides with proteases and synthesized l-carnitine using lysine and methionine [25,37].

### 2.3. Antioxidant Activity of Fermented Ginseng by R. oligosporus

There are two major mechanisms for antioxidant activity present in plant deactivation of radicals by hydrogen atom transfer (HAT) and by single electron transfer (SET) [38]. Therefore, we selected oxygen radical absorbance capacity (ORAC) utilizing the HAT reaction, ferric reducing antioxidant powder (FRAP) utilizing the SET reaction and total phenolic contents utilizing both HAT reaction and SET reaction to determine antioxidant activity of the fermented wild ginseng [38]. 

#### 2.3.1. Total Phenolic Content

**Figure 3:**
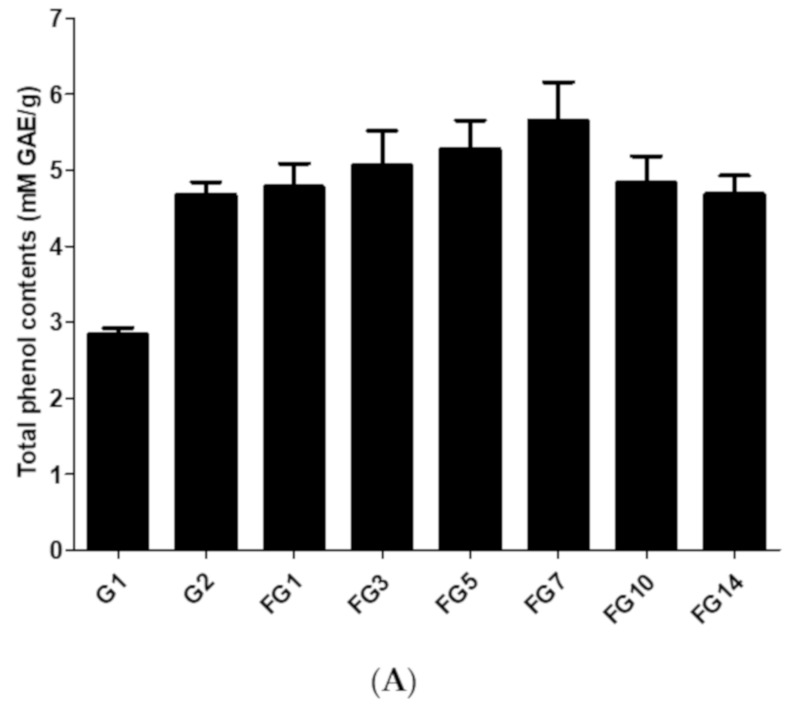

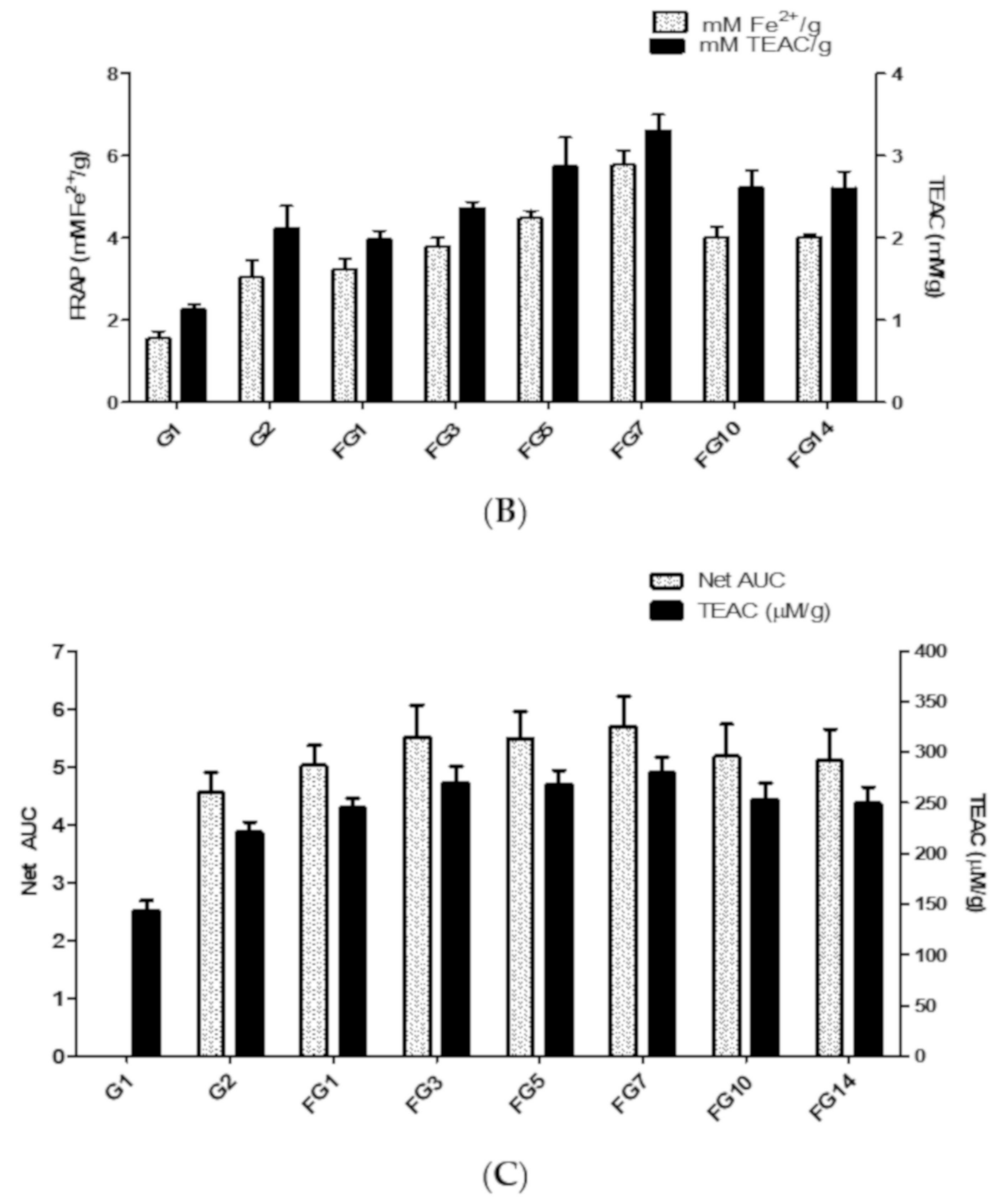
Total phenol content (**A**), ferric reducing antioxidant power (FRAP, **B**) and oxygen radical absorbance capacity (ORAC, **C**) of fermented wild ginseng by *R. oligosporus*. G1: wild ginseng extract; G2: autoclaved wild ginseng extract; FG1: 1-day fermented wild ginseng extract; FG3: 3-day fermented wild ginseng extract; FG5: 5-day fermented wild ginseng extract; FG7: 7-day fermented wild ginseng extract; FG10: 10-day fermented wild ginseng extract and FG14: 14-day fermented wild ginseng extract. All experiments were performed in triplicate and all data were expressed as the mean ± standard deviation. TEAC: Trolox equivalent antioxidant capacity.

The total phenolic contents of extracted wild ginseng, autoclaved wild ginseng and fermented wild ginseng over a period of 1–14 days are shown in Figure 3A. The total phenolic contents increased from 2.84 ± 0.12 mM GAE/g in wild ginseng extract to 4.68 ± 0.23 mM GAE/g in autoclaved wild ginseng extract and further increased to 5.65 ± 0.72 mM GAE/g in the 7-day fermented wild ginseng extract (Figure 3A). Then the total phenolic contents were decreased to 4.69 ± 0.34 mM GAE/g in the 14-day fermented wild ginseng extract. The highest total phenolic content of fermented wild ginseng extract was obtained after 7-day fermentation. 

#### 2.3.2. Ferric Reducing Antioxidant Power (FRAP)

The FRAP assay is based on the measurement of the ability of ginseng extract reducing ferric tripyridyltriazine (Fe^3+^-TPTZ) to a blue-colored product (ferrous-tripyridyltriazine, Fe^2+^-TPTZ) [39]. The ferric reducing power of extracted wild ginseng, autoclaved wild ginseng and fermented wild ginseng from 1–14 days are shown in Figure 3B. The ferric reducing power increased from 1.55 ± 0.18 mM Fe^2+^/g (or 1.13 ± 0.06 mM TEAC/g) in wild ginseng extract to 5.76 ± 0.36 mM Fe^2+^/g (or 3.31 ± 0.19 mM TEAC/g) in 7-day fermented wild ginseng extract. Then the reducing power was decreased to 4.02 ± 0.06 mM Fe^2+^/g (or 2.60 ± 0.20 mM TEAC/g) in 14-day fermented wild ginseng extract. A Pearson correlation test was conducted to determine the relationship between the total phenolic contents and the reducing power. The total phenolic contents of fermented ginseng correlated with FRAP (*r* = 0.912) indicated that the total phenolic contents and FRAP of fermented wild ginseng have a very strong positive correlation.

#### 2.3.3. Oxygen Radical Absorbance Capacity (ORAC)

The ORAC assay is based on the measurement of the antioxidant capacity of the ginseng extract to inhibit the peroxyl radical-induced oxidations [40]. Trolox was used as a standard for the ORAC assay (Figure S1). The results of an ORAC assay performed in this study are shown in Figure 3C. As shown in Figure 3C, the antioxidant capacity of fermented wild ginseng increased with increasing fermentation time up to 7 days (279.32 ± 15.53 μM trolox/g) and then decreased after 14-day fermentation (249.6 ± 16.07 μM trolox/g). A Pearson correlation test was conducted to determine the relationship between the total phenolic contents and ORAC. The total phenolic contents of fermented wild ginseng correlated with ORAC (r = 0.974), indicating that the total phenolic contents and ORAC of fermented wild ginseng have a very strong positive correlation. 

### 2.4. Inhibitory Effect of Fermented Ginseng against Nitric Oxide Production

**Figure 4:**
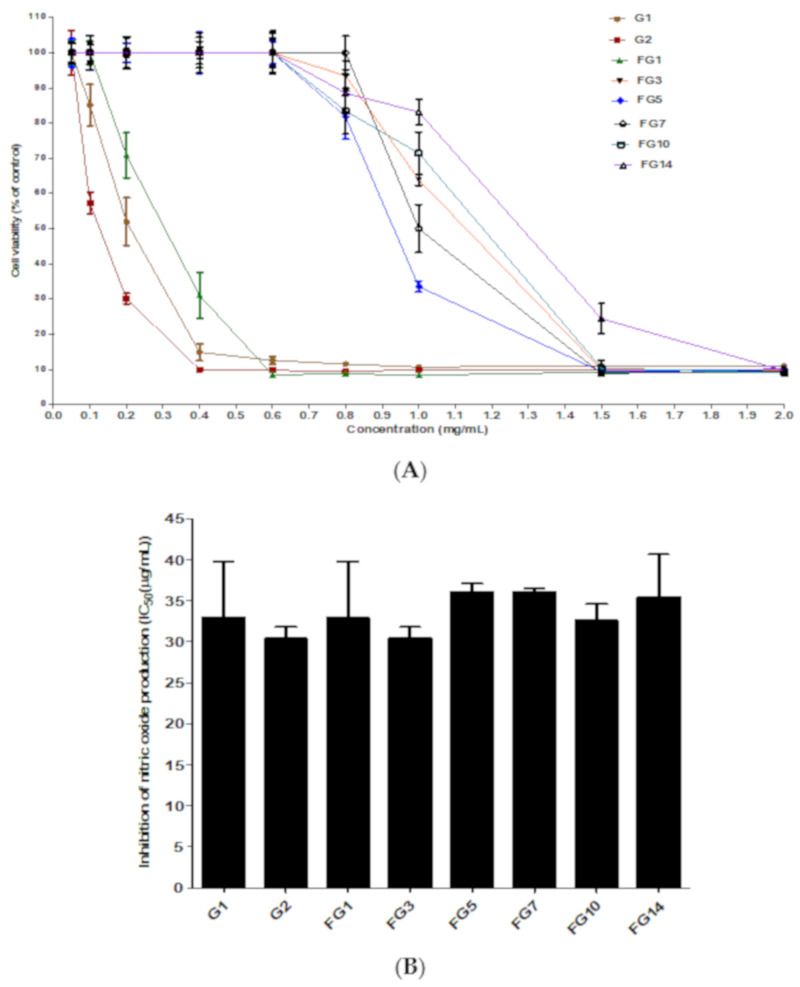
Cell viability (**A**) and nitric oxide inhibition activity (**B**) of fermented wild ginseng by *R. oligosporus* on RAW264.7 murine macrophage cells. (**A**,**B**) (**●, G1**): wild ginseng extract; (**○**, G2): autoclaved wild ginseng extract; (**▼**, FG1): 1-day fermented wild ginseng extract; (**∆**, FG3): 3-day fermented wild ginseng extract; (**■**, FG5): 5-day fermented wild ginseng extract; (**□**, FG7): 7-day fermented wild ginseng extract; (**◆**, FG10): 10-day fermented wild ginseng extract and (**◇**, FG14): 14-day fermented wild ginseng extract. All experiments were performed in triplicate and all data were expressed as the mean ± standard deviation.

Inflammation is a biological defense mechanism in the human body activated in response to invading pathogens and other danger signals. The lipopolysaccharides (LPS)-stimulated RAW264.7 macrophages cells are usually selected to investigate the anti-inflammatory activity of natural products and herbal medicine due to their sensitivity to LPS stimulation and release of different kinds of inflammatory mediators such as tumor necrosis factor-alpha, interleukin and nitric oxide (NO) [41]. Among them, the reactive free radical NO made by inducible nitric-oxide synthase (iNOS) is one of the important inflammatory mediators, known to participate in the occurrence of a number of inflammatory diseases [42,43]. First, the RAW264.7 cell cytotoxicity of wild ginseng, autoclaved wild ginseng and fermented wild ginseng was investigated. The cell viabilities of RAW264.7 murine macrophage cells compared to cells without a treated ginseng sample are shown in Figure 4A. The cell viability changed depending on the fermentation time. The cell viability at 0.05, 0.1, 0.2, 0.4, 0.6, 0.8 and 1 mg/mL was 100% ± 4.1%, 85.1% ± 4.1%, 52.0% ± 6.9%, 14.8% ± 2.4%, 12.6% ± 1.0%, 11.5% ± 0.5% and 10.7% ±0.3% for wild ginseng, 100% ± 6.3%, 57.3% ± 3.1%, 30.1% ± 1.5%, 10.0% ± 0.1%, 9.8% ± 0.2%, 9.4% ± 0.1% and 10.0% ± 0.2% for autoclaved wild ginseng, 100% ± 3.6%, 100% ± 3.4%, 70.9% ± 7.4%, 31.3% ± 6.5%, 8.5% ± 0.2%, 8.8% ± 0.2% and 8.3% ± 0.3% for 1-day fermented wild ginseng, respectively. The fermented wild ginseng extract after 3–14 days of fermentation showed 100% cell viability up to concentration of 0.6 mg/mL and over 80% cell viability at 0.8 mg/mL. The cell viability of fermented ginseng after 3, 5, 7, 10 and 14-days at 1 mg/mL was 63.9% ± 1.6%, 33.6% ± 1.6%, 50.0% ± 6.8%, 71.5% ± 6.1% and 83.2% ± 3.7%, respectively. Cell viability at 1.5 mg/mL of wild ginseng, autocleaved wild ginseng and fermented wild ginseng at 1, 3, 5, 7, 10 and 14-day was from 8.8% ± 0.3% to 24.4% ± 4.3%. From these results, the fermented wild ginseng extract showed reduced cytotoxicity with respect to the wild and autoclaved extracts. Based on these results, the nitric oxide assay was conducted at 0.06 mg/mL. The anti-inflammatory ability of wild ginseng (with/or without being autoclaved) and fermented wild ginseng after 1, 3, 5, 7, 10 and 14 days by evaluating their inhibitory effects on NO synthesis in LPS-stimulated RAW264.7 cells are shown in Figure 4B. LPS led to an increase in NO production (8.1 µM NO) compared with the negative control (0 µM NO; data not shown), but all of the ginseng extracts at concentrations of 10, 20, 40 and 60 µg/mL caused dose-dependent reductions in the NO production compared with NO production in LPS-stimulated RAW264.7 cells. The 50% inhibitory concentration (IC_50_) of wild ginseng, autoclaved wild ginseng and fermented wild ginseng (1, 3, 5, 7, 10 and 14 day) against NO production in LPS-induced RAW264.7 are 33.0 ± 6.9, 30.5 ± 1.3, 33.0 ± 6.9, 30.5 ± 1.3, 36.1 ± 0.8, 36.1 ± 0.4, 32.6 ± 1.6 and 35.5 ± 4.3 μg/mL, respectively. There was no significant difference among their inhibitory activities against NO production in LPS-induced RAW264.7. The anti-inflammatory activity of ginsenosides on RAW264.7 cells due to ginsenosides are regulated inflammatory responses primarily through the inhibition of the NF-κB signaling pathway. In LPS-stimulated macrophages and microglial cells, ginsenosides suppress the production of proinflammatory cytokinases such as TNF-α, IL-1β and IL-6 as well as inflammatory enzymes such as iNOS and COX-2 [44]. 

## 3. Materials and Methods

### 3.1. Materials

Wild ginseng (4 years old; *P. ginseng*) roots were purchased from a local market (Pyeongchang, Korea). A wild ginseng sample was lyophilized at −40 °C at 10 Pa for 3 days, then pulverized and stored at −20 °C for further study. Eighteen ginsenoside standards (Rb1, Rb2, Rb3, Rg1, Rg2, Rg3, Rc, Rd, Re, Rf, Ro, R1, R2, Rh1, Rh2, F1, F2 and compound K) were purchased from ChemFaces (Wuhan, China). Other materials including 2,2-diphenyl-1-picrylhydrazyl (DPPH), Folin–Ciocalteu reagent, 6-hydroxy-2,5,7,8-tetramethylchromane-2-carboxylic acid (trolox), 2,2’-Azobis(2-methylpropionamidine) dihydrochloride, lipopolysaccharides and gallic acid were bought from Sigma-Aldrich (St. Louis, MO, USA). Fluorescein was purchased from Alfa Aesar (Haverhill, MA, USA). Acetonitrile and ethyl alcohol (HPLC-grade) were obtained from Honeywell—Burdick and Jackson (Muskegon, MI, USA). Formic acid was purchased from Thermo Fisher Scientific Korea LTD (Seoul, Korea). RAW264.7 murine macrophage cells were bought from Korean Cell Line Bank (Seoul, Korea). Cell cultures were maintained in Dulbecco’s modified Eagles medium (DMEM), fetal bovine serum (FBS) was purchased from Gene depot (Barker, TX, USA) and penicillin and streptomycin were purchased from Invitrogen (Carsbad, CA, USA). For cell viability assays, Ez-CyTox solution was purchased from Daeil Lab Service (Seoul, Korea).

### 3.2. Fermentation of Wild Ginseng by R. oligosporus

*R. oligosporus* was isolated from our previous study [26,27,37] and cultured on potato dextrose agar (PDA, Difco, Detroit, MI, USA) medium at 30 °C for 3 days to obtain spores. Wild ginseng powder was sterilized at 121 °C for 15 min. Fermentation was done by inoculating 8.0 × 10^5^ spores/g of *R. oligosporus* to wild ginseng powder at 30 °C for 1–14 days. Then, fermented wild ginseng was frozen at −80 °C and lyophilized at 0 °C at 10 Pa. Wild ginseng without fermentation was used as a control and prepared as described above.

### 3.3. Sample Extraction

One and half grams of wild ginseng, autoclaved wild ginseng and fermented wild ginseng were extracted with 30 mL of 60% ethanol at 200 rpm, 40 °C for 1 h. The process was repeated three times, followed by filtration through Whatman no. 1 filter paper (Piscataway, NJ, USA). Then the ethanol was removed by evaporation (Heidolph Instruments, Schawabatch, Germany). The extracted sample was lyophilized. The extraction yield was calculated according to Equation (1):

(1) $$\mathrm{Yield}\;(\%)=\frac{extract\;mass\;\left(g\right)}{wild\;cultivated\;ginseng\;powder\;\left(g\right)}\times 100$$

### 3.4. Analysis of Ginsenoside Contents

Twenty milligrams of extracted wild ginseng, auto-cleaved wild ginseng and fermented wild ginseng was dissolved in 5 mL of distilled water and purified using Phenomenex strata C_18_ Cartridge (Phenomenex, Inc., Torrance, CA, USA). The cartridge was activated with 2 mL of methanol followed by 2 mL of distilled water. One milliliter of sample was filtered through a cartridge and washed with 2 mL of distilled water. The cartridge was eluted with 2 mL of methanol and filtered using a 0.2 μm Minisart^®^ syringe filter (Goettingen, Germany). One microliter of sample was injected to the UPLC–MS system (Waters Acquity H-Class system with Waters QDa detector, Waters, Milford, MA, USA). The separation was conducted on a Waters BEH C_18_ 1.7 μm × 2.1 mm × 100 mm column at 40 °C. The mobile phase consisted of acetonitrile (solvent A) and 0.1% formic acid in water (solvent B). The elution gradient used was as follows: 5% A initially, increased to 33% A at 2 min, 38% A at 9 min, 100% A at 16 min, 5% A at 16.1 min and maintained until 20 min for equilibrium step with flow rate 0.3 mL/min. Each of the 14 detected ginsenosides was subjected to electrospray ionization (ESI; positive and negative) with selective ion recording (SIR; Table S1). The calibration curves for ginsenosides were prepared using the internal standard method for the 14 ginsenosides at various concentrations (Table S2). The linear correlation between standard concentrations and area was evaluated (r^2^ > 0.99). The limits of detection (LOD) and quantitation (LOQ) were determined via the linear regression method [45]. The LOD and LOQ can be expressed as: LOD = 3Sa/b, LOQ = 10 Sa/b, where Sa denotes the standard deviation of the response and b is the slope of the calibration curve of each standard. Fourteen ginsenoside standards were prepared at low concentration (0.02–0.2 μg/mL) and analyzed 7 times. The LOD and LOQ values ranged from 0.007 to 0.059 μg/mL and 0.022 to 0.196 μg/mL, respectively (Table S2).

### 3.5. Analysis of l-Carnitine Content

The l-carnitine content in fermented wild ginseng extracts was analyzed using the method of Park et al. with slight modification [37]. Fermented wild ginseng extract (10 mg) was dissolved in 1 mL of methanol and filtered through a 0.2 μm syringe filter (Satorius AG, Goettingen, Germany). One microliter of sample was injected into the UPLC–MS system. The separation was achieved on a Waters BEH HILIC 1.7 μm × 2.1 mm × 100 mm column at 40 °C. The mobile phase consisted of acetonitrile with 0.1% formic acid (solvent A) and 15 mM ammonium formate with 0.1% formic acid in water (solvent B). The elution gradient used was as follows: 10% B initially, increased to 30% B at 5 min, 60% B at 6 min and maintained until 10 min for equilibrium step. Electrospray ionization (ESI) was positive with single ion recording (SIR; l-carnitine: 162 *m*/*z*, Table S1). An external standard method was used for the quantification of l-carnitine in fermented ginseng sample. A linear relationship between the standard concentrations and area was evaluated (r^2^ > 0.99; Table S2).

### 3.6. Antioxidant Activity

#### 3.6.1. Determination of Total Phenolic Contents

The determination of total phenolic contents was carried out according to the method of Folin–Ciocalteu [27], with gallic acid (GAE) as the standard. The total phenolic contents of the sample were presented as mM GAE/g dry sample.

#### 3.6.2. Ferric Reducing Antioxidant Power (FRAP) Assay

The FRAP assay of wild or fermented wild ginseng extract was carried out as described by Jang et al. with slight modification [46]. The FRAP solution was prepared by using 0.3 M sodium acetate buffer (pH 3.6), 10 mM 2,4,6-tripyridyl-s-triazine in 40 mM HCl and 20 mM ferric chloride hexahydrate solution with a ratio 10:1:1 (*v*/*v*/*v*) at 37 °C. The sample (20 μL), trolox (0–40 μM) as standard control, or varied concentration of ferric sulfate (1–200 μM) was mixed with 180 μL FRAP solution. After 30 min incubation at room temperature in the dark, the sample absorbance was read at 593 nm (SpectraMax M3, Molecular Devices, Synnyvale, CA, USA). The results were expressed as mM Fe^2+^/g and mM TEAC/g.

#### 3.6.3. Oxygen Radical Absorbance Capacity (ORAC) Assay

The ORAC assay of wild ginseng, autoclaved wild ginseng and fermented wild ginseng after 1–14 days was carried out according to the method by Ou et al. [40]. Briefly, 10 μL of sample or trolox (0–40 μM) as a standard control was incubated with 90 μL 25 nM fluorescein and 100 μL 25 mM AAPH in a total volume of 200 μL. The fluorescence of reaction mixtures was measured every 3 min (λ_excitation_ = 485 nm, λ_emission_ = 538 nm) up to 2 h at constant 37 °C with SpectraMax M3. Trolox was used as the standard compound. The net area under the curve (net AUC) was calculated by subtracting the AUC of the blank from the AUC of each tested sample. The antioxidant capacity was quantified as a Trolox equivalent.

### 3.7. Cell Viability Test

RAW264.7 murine macrophage cells grown in 96-well plate at 2 × 10^4^ cells/well were treated with different sample concentrations (0.05–2.0 mg/mL) for 24 h at 37 °C and 5% CO_2_. Then 10 μL of Ez-CyTox solution was mixed with 90 μL of medium and incubated for 1 h at 37 °C. The optical density (OD) was detected at 450 nm using a SpectraMax M3, and the cell viability was calculated as a percentage of the control [27].

### 3.8. Measurement of Nitric Oxide Production

RAW264.7 cells grown on 96-well plate at 2 × 10^4^ cells/well for 48 h were treated with different sample concentrations (5–60 μg/mL) with 1 μg lipopolysaccharide (LPS)/mL at 37 °C for 24 h. Cells treated with 1 μg/mL LPS and 100 μM indomethacin were used as control. Eighty microliters of culture supernatant was mixed with an equal volume of Griess reagent for 20 min. The absorbance was measured at 540 nm using a SpectraMax M3, and the NO concentration in the sample was calculated from a sodium nitrite standard curve (0–500 μM in cell culture medium) [27].

### 3.9. Statistical Analysis

All experiments were performed in triplicate, and all data were expressed as the mean ± standard deviation. The data were analyzed by a one-way ANOVA and Duncan’s multiple range test to determine significant differences among means. A Pearson correlation test was conducted to analyze the relationship between total phenolic contents and FRAP or ORAC. All statistical analyses were accomplished using the IBM SPSS Statistics for Windows, version 25 (IBM Crop., Armonk, NY, USA).

## 4. Conclusions

Our studies demonstrated, for the first time, that l-carnitine is produced in wild ginseng extract after fermentation using *R. oligosporus*. The fermented wild ginseng extracts contained increased amounts of the total ginsenosides (especially Rd) and total phenolic contents. These compounds enhanced the antioxidant capacity and anti-inflammatory activity of fermented ginseng extract compared with that of the wild variety. Therefore, this study suggests the potential application of the fermented ginseng in the food and pharmaceutical industry.

## Figures and Tables

**Table 1:** Extraction yields of fermented wild ginseng.

	Control	Autoclaved-Control	Fermented Wild Ginseng					
			1 day	3 days	5 days	7 days	10 days	14 days
**Extraction Yield (%)**	21.2	20.7	21.4	23.0	23.8	24.9	23.3	25.4

All experiments were performed in triplicate and all data were expressed as the mean ± standard deviation.

**Table 2:** Ginsenoside contents in the fermented wild ginseng following *R. oligosporus* fermentation.

Group	Chemicals	Ginsenoside Contents (mg/Kg)							
		Control *	Autoclaved Control	Fermented Wild Ginseng					
				1 day	3 days	5 days	7 days	10 days	14 days
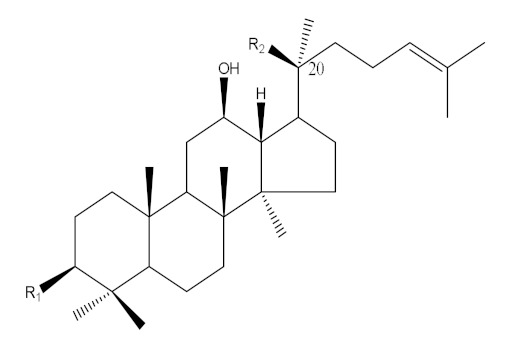 **Protopanaxadiol-type (PPD)**	**Rg3**	70 ^a^	100 ± 10 ^e^	80 ± 10 ^e^	140 ± 10 ^c^	160 ± 10 ^d^	170 ± 10 ^de^	180 ^e^	230 ± 10 ^f^
	**Rb1**	4030 ± 540 ^c^	7890 ± 1240 ^d^	7840 ± 810 ^d^	4300 ± 210 ^c^	2380 ± 90 ^b^	3370 ±120 ^c^	410 ± 10 ^a^	400 ± 40 ^a^
	**Rb2**	1410 ± 210 ^a^	2810 ± 410 ^c^	2770 ± 120 ^c^	2300 ± 160 ^b^	2850 ± 80 ^c^	2810 ± 200 ^c^	2660 ± 330 ^bc^	3730 ± 230 ^d^
	**Rb3**	220 ± 40 ^a^	420 ± 20 ^cd^	310 ± 30 ^b^	380 ± 50 ^bc^	460 ± 50 ^d^	470 ± 50 ^d^	490 ± 60 ^d^	580 ± 20 ^e^
	**Rc**	2130 ± 130 ^a^	3800 ± 420 ^bc^	3660 ± 170 ^bc^	3220 ± 120 ^b^	4060 ± 540 ^c^	4150 ± 650 ^c^	3560 ± 320 ^bc^	5280 ± 500 ^d^
	**Rd**	1280 ± 60 ^a^	2020 ± 370 ^a^	2140 ± 100 ^a^	4870 ± 330 ^b^	6940 ± 910 ^cd^	6730 ± 300 ^c^	7760 ± 810 ^d^	11,300 ± 840 ^e^
	**Compound K**	20 ^a^	50 ^d^	40 ^b^	40 ^c^	50 ^d^	60 ^e^	70 ^f^	70 ^g^
	**F2**	110 ± 10 ^a^	150 ± 20 ^b^	110 ± 10 ^ab^	190 ± 10 ^c^	240 ± 20 ^d^	260 ± 20 ^d^	240 ± 20 ^d^	310 ± 30 ^e^
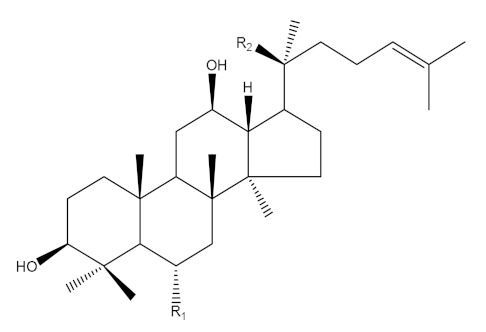 Protopanaxatriol-type (PPT)	**Rg1**	6330 ± 340 ^a^	7060 ± 810 ^a^	8060 ± 470 ^bc^	6440 ±340 ^a^	7240 ± 670 ^ab^	7120 ± 280 ^ab^	6520 ± 340 ^a^	8950 ± 710 ^c^
	**Rg2**	850 ± 30 ^a^	1560 ± 50 ^bc^	1340 ± 60 ^b^	1320 ± 30 ^b^	1500 ± 90 ^c^	1850 ± 30 ^e^	1630 ± 40 ^e^	2050 ± 80 ^f^
	**Re**	9790 ± 340 ^a^	10,420 ± 1580 ^a^	12,500 ± 480 ^bc^	9900 ± 660 ^a^	11,330 ± 1380 ^ab^	10,740 ± 410 ^ab^	9750 ± 510 ^a^	13,730 ± 1560 ^c^
	**Rf**	6410 ± 220 ^a^	6870 ± 660 ^a^	7860 ± 640 ^bc^	6370 ± 390 ^a^	7110 ± 640 ^ab^	7120 ± 390 ^ab^	6460 ± 300 ^a^	8560 ± 570 ^c^
	**Rh1**	60 ± 10 ^a^	310 ^d^	170 ^b^	200 ± 20 ^b^	260 ± 10 ^c^	400 ± 40 ^e^	340 ± 20 ^d^	410 ± 20 ^e^
	**F1**	40 ^a^	110 ^c^	100 ± 10 ^bc^	90 ± 10 ^b^	100 ± 10 ^bc^	110 ± 10 ^c^	100 ± 10 ^bc^	140 ^d^

All experiments were performed in triplicate, and all data were expressed as the mean ± standard deviation. The different letters of the alphabet in each row represent significant differences (*p* < 0.05). * Control: wild ginseng extract.

**Table 3:** l-carnitine contents in fermented wild ginseng.

	Control *	Autoclaved-Control	Fermented Wild Ginseng					
			1 day	3 days	5 days	7 days	10 days	14 days
l **-carnitine (mg/kg)**	ND *	ND *	60 ^a^	230 ^b^	310 ^c^	440 ^d^	500 ^e^	630 ^f^ ± 10

All experiments were performed in triplicate, and all data were expressed as the mean ± standard deviation. The different letters of alphabet in each row represent significant differences (*p* < 0.05). * Control: wild ginseng extract. ND. Not detected.

## Supplementary Materials

The following are available online, Figure S1: Oxygen radical absorbance capacity (ORAC) of Trolox at different concentration, Table S1: LC-QDa mass condition for ginsenoside and L-carnitine (SIR), Table S2: Summary of qualification results for linearity, limit of detection (LOD), and limit quantification (LOQ) of 14 ginsenosides and L-carnitine.
