# Supplementary material for: Fermented Wild Ginseng by Rhizopus oligosporus Improved l-Carnitine and Ginsenoside Contents
Source: Molecules. 2020 Apr 30;25(9):2111. doi: 10.3390/molecules25092111 (PMC7249200; doi:10.3390/molecules25092111)
Supplement: Supplementary file 1 [file molecules-25-02111-s001.pdf]

molecules MDPI

Table S1. LC-QDa mass condition for ginsenoside and L-carnitine (SIR).

No.	Name	MW	M/Z	Corn voltage	Retention time (min)
1	Rb1	1,109.3	1,132.0 [M+Na]+	10	7.434
2	Rb2	1,079.3	1,101.6 [M+Na]+	15	8.997
3	Rb3	1,079.3	1,101.6 [M+Na]+	15	9.234
4	Rc	1,079.3	1,101.6 [M+Na]+	35	8.142
5	Rd	947.2	969.7 [M+Na]+	20	11.137
6	Rg3	785.0	807.6 [M+Na]+	50	15.138
7	F2	785.0	829.7 [M+HCOO]-	10	14.255
8	Compound K	622.9	645.5 [M+Na]+	45	16.421
9	Rg1	801.0	823.6 [M+Na]+	25	4.498
10	Rg2	785.0	783.6 [M-H]-	40	8.258
11	Re	947.2	945.6 [M-H]-	45	4.453
12	Rf	801.0	823.6 [M+Na]+	10	6.978
13	Rh1	638.9	683.5 [M+HCOO]-	20	8.488
14	F1	638.9	661.6 [M+Na]+	25	10.48
15	L-carnitine	161.2	162.0 $[M+H]^+$	10	5.04

Table S2. Summary of qualification results for linearity, limit of detection (LOD), and limit of quantification (LOQ) of 14 ginsenosides and L-carnitine**.**

Chemicals	Concentration range (µg/ml)	Linearity (R^2)	Regression equation	LOD (µg/ml)	LOQ (µg/ml)
Rb1	0.08-1.2	0.9953	Y=0.0349X-0.0008	0.059	0.196
Rb2	0.08-1.0	0.9918	Y=0.0854X-0.0055	0.039	0.129
Rb3	0.08-1.2	0.9932	Y=0.0956X-0.0063	0.040	0.135
Rc	0.04-1.2	0.9962	Y=0.0884X-0.0032	0.028	0.095
Rd	0.04-1.0	0.9922	Y=0.1103X-0.0040	0.049	0.165
Rg3	0.02-1.8	0.9955	Y=0.4755X-0.0234	0.033	0.109
F2	0.02-1.8	0.9962	Y=0.1075X-0.0017	0.007	0.022
Compound K	0.02-1.8	0.9985	Y=1.9225X-.0444	0.007	0.023
Rg1	0.04-1.8	0.9962	Y=0.4856X+0.0059	0.012	0.040
Rg2	0.02-1.8	0.9984	Y=0.1572X-0.0022	0.009	0.030
Re	0.02-1.8	0.9975	Y=0.0829X+0.0000	0.013	0.044
Rf	0.02-1.8	0.9968	Y=0.4885X+0.0086	0.009	0.031
Rh1	0.02-1.8	0.9978	Y=0.1975X-0.0031	0.004	0.014
F1	0.04-1.8	0.9916	Y=0.6326X-0.0315	0.023	0.076
L-carnitine	0.01-1.0	0.9999	$Y=3.51\times10^7X+44600$	0.008	0.024

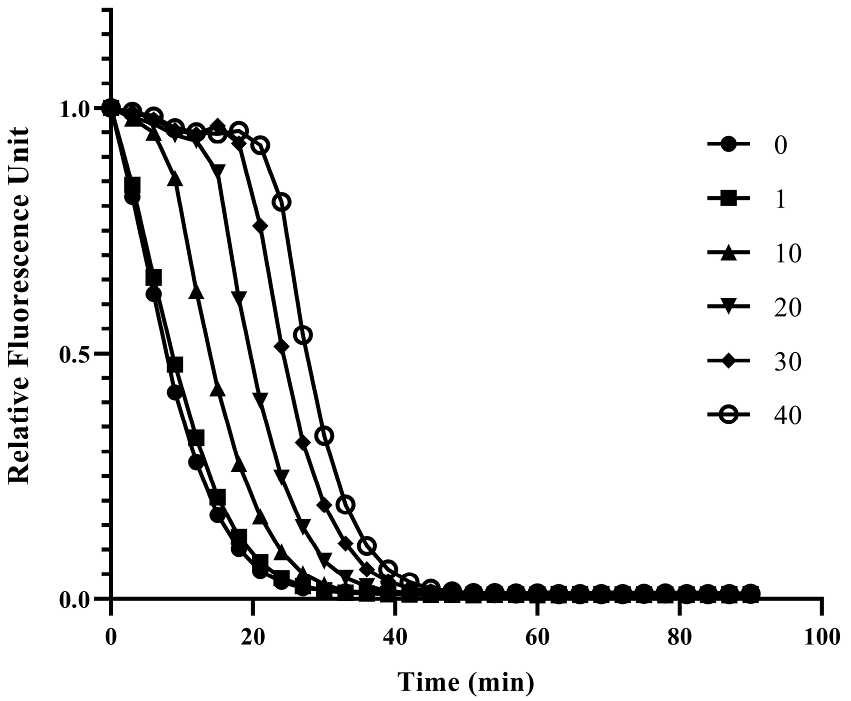

Figure S1. Oxygen radical absorbance capacity (ORAC) of Trolox at different concentration. (●): 0 μM; (■): 1 μM; (▲): 10 μM; (▼): 20 μM; (◆): 30 μM; (○): 40 μM;.
